# Segmental differences of cervical spinal cord motion: advancing from confounders to a diagnostic tool

**DOI:** 10.1038/s41598-019-43908-x

**Published:** 2019-05-15

**Authors:** M. Hupp, K. Vallotton, C. Brockmann, S. Huwyler, J. Rosner, R. Sutter, M. Klarhoefer, P. Freund, M. Farshad, A. Curt

**Affiliations:** 1 Spinal Cord Injury Center, Balgrist University Hospital, Zurich, Switzerland; 2 Radiology, Balgrist University Hospital, Zurich, Switzerland; 3 Siemens Healthcare AG, Zurich, Switzerland; 4 University Spine Centre Zurich, Balgrist University Hospital, Zurich, Switzerland

**Keywords:** Prognostic markers, Magnetic resonance imaging, Neurology, Spinal cord diseases

## Abstract

Increased cranio-caudal spinal cord motion is associated with clinical impairment in degenerative cervical myelopathy. However, whether spinal cord motion holds potential as a neuroimaging biomarker requires further validation. Different confounders (i.e. subject characteristics, methodological problems such as phase drift, etc.) on spinal cord motion readouts have to be considered. Twenty-two healthy subjects underwent phase contrast MRI, a subset of subjects (N = 9) had repeated scans. Parameters of interest included amplitude of velocity signal, maximum cranial respectively maximum caudal velocity, displacement (=area under curve of the velocity signal). The cervical spinal cord showed pulse synchronic oscillatory motions with significant differences in all readouts across cervical segments, with a maximum at C5. The Inter-rater reliability was excellent for all readouts. The test-retest reliability was excellent for all parameters at C2 to C6, but not for maximum cranial velocity at C6 and all readouts at C7. Spinal cord motion was correlated with spinal canal size, heart rate and body size. This is the first study to propose a standardized MRI measurement of spinal cord motion for further clinical implementation based on satisfactory phase drift correction and excellent reliability. Understanding the influence of confounders (e.g. structural conditions of the spine) is essential for introducing cord motion into the diagnostic work up.

## Introduction

Oscillatory spinal cord motions were initially shown by intraoperative ultrasound^1^. Later on, phase-contrast MRI (PC-MRI) allowed assessments of *in vivo* spinal cord motions and cerebrospinal fluid (CSF) dynamics. Cardiac-gated velocity measurements can be mapped over the heart cycle resulting in a flow-profile^2,3^. In patients with degenerative cervical myelopathy (DCM), several studies applying 2D PC-MRI reported increased spinal cord motion at the level of a cervical spinal stenosis^4–7^. A better insight into pathophysiological processes contributing to spinal cord dysfunction is fundamental for understanding different disease stages that may be less appreciable when only relying on static anatomic measures. In addition, conventional MRI provides only static information, while PC-MRI evaluates dynamic mechanical factors which might contribute to the development of cervical myelopathy^8,9^. Interestingly, increased spinal cord motion was associated with sensory deficits^4,6^, impaired electrophysiological readouts^6^ and decreased functional scores in DCM patients^7^. Thus, altered spinal cord motion might be a potential surrogate of spinal cord dysfunction. While measurements of CSF flow have been shown to be less reliable and rather complex (i.e. not easy to implement and run for clinical application) at the level of stenosis^7^, spinal cord motion appears as an attractive alternative. The latter is easy to apply and provides parameters that potentially allow to evaluate the extent of cervical dysfunction and could eventually be used to predict the course of DCM. Cervical spinal cord motions have been evaluated less systematically across the cervical spinal segments. The impact of anatomic (i.e. spinal canal size) and dynamic factors (i.e. blood pressure, heart rate) remains unknown. Previous studies report different parameters of spinal cord motions^4,6,7^ and therefore are not readily comparable. Additionally, PC MRI readouts are influenced by a varying, so called “phase drift”^10^, ending in false velocity values, caused by a baseline offset error. Several reasons have been proposed for the observed errors including acceleration artefacts and phase contributions^11^, flow related eddy current effects^12,13^, maxwell gradient effects^14^, voxel size related partial-volume effects^15^, relaxation effects^16^, phase errors during the time between velocity encoding and echo, intravoxel velocity distribution^17^ and other non-identified effects^13^. Standardized correction for phase drift is needed as it varies between scanners and different timepoints of measurement^10^ and was proposed in diverse ways (phantom measurements^2,5,18^, measurements of static tissue^18^). However, no standardized measurement of spinal cord motion is established yet.

Therefore, a reliable and sensitive assessment of cervical spinal cord motions along the cervical spine is required in order to foster its clinical application and potential introduction in the diagnostic work up of DCM.

The aim of this study was to investigate cranio-caudal spinal cord motions across the entire cervical spine in healthy volunteers, to evaluate its inter-rater and test-retest reliability for clinical application and its relation to anatomic and biometric conditions.

## Methods

### Population

This prospective study (2016–2018) was approved by the local ethics committee (Kantonale Ethikkommission Zurich, KEK-ZH 2012-0343, BASEC Nr. PB_2016-00623). All methods were carried out in accordance with the relevant guidelines and regulations. Informed consent was obtained from all participants.

22 healthy subjects (age 63.3 ± 6.6 years) were recruited. Four subjects with incidental stenosis in structural imaging had to be excluded. Eighteen participants (9m, 9f; age 62.2 ± 6.5 years) were included in the final statistical analysis. Data on blood pressure before and after the MRI, age, body weight and body size were collected. Blood pressure, weight and body size was missing in one participant each.

### Imaging

**Figure 1:**
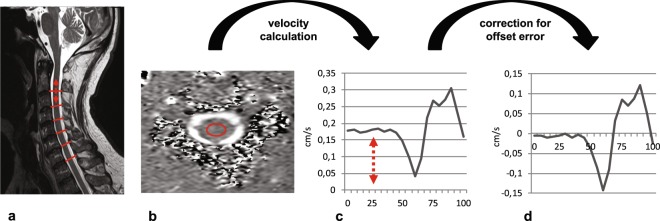
Steps in spinal cord motion evaluation. Spinal cord cranio-caudal motion was measured by axial MRI phase contrast imaging in each cervical segment (**a**; sagittal T2 image; red lines identifying the cervical segments; arrow illustrating motion). Motion was encoded into grey values of the MRI image within 20 timepoints of one heartcycyle. Velocity values were calculated by the mean of collected grey value within the predefined region of interest (**b**; axial phase contrast image; red circle illustrating predefined ROI) and the used velocity encoding in the sequence (2 cm/s). Due to a technically caused offset error (**c**; uncorrected motion signal during one heartcycle (0–100%); arrow indicating offset error) raw velocity values were misleading overestimated. Subtracting the mean of all 20 velocity values from each single value could sufficiently correct for the offset error (**d**; corrected motion signal during one heartcycle (0–100%)). Readouts of spinal cord motion used were amplitude of the motion signal, ranging from the maximum negative to the maximum positive value, maximum cranial velocity, maximum caudal velocity and the displacement (=area under curve of velocity signal).

All subjects underwent a MRI scan (3T, MAGNETOM SkyraFit, Siemens Healthcare, Erlangen) including sagittal and axial standard clinical T2w sequences and axial 2D phase contrast imaging encoding cranio-caudal spinal cord motion in each cervical segment (Fig. 1a). Slice orientation in phase contrast imaging was adjusted perpendicular to the spinal cord. The phase contrast sequence was cardiac gated through peripheral pulse triggering using a finger clip. The venc (velocity encoding) value of the phase contrast sequence was set to 2 cm/s based on previous findings of cord motion^4–7,18^. During one cardiac cycle velocity signal was assessed within 20 time points and 128 measurements were averaged per segment. Total scanning time of the whole protocol was 23 min.

### Imaging analysis

Image analysis was performed using the OsirixTM free DICOM viewer (www.osirix.com).

### Anatomical measurements

In sagittal T2w images the anterior-posterior diameter (ap) of the spinal canal at disc level, in axial images the anterior-posterior (ap) and right-left (rl) diameter, as well as spinal canal and spinal cord cross-sectional area (CSA) at disc level were measured in all cervical segments. To evaluate the apex of the cervical spine curvature tangential lines were drawn at the dorsal vertebral bodies with the point of intersection identifying the apex and additionally the angle was collected.

### Velocity calculation

In phase contrast measurements, cranio-caudal spinal cord motion was analysed by a predefined ellipsoid shaped region of interest (30,52 mm^2^) mid-centred into the spinal cord (Fig. 1b). According to the predefined velocity encoding (2 cm/s) of phase contrast imaging velocity signal within the MRI sequence ranges from −2 cm/s to 2 cm/s encoded in grey values from −4096 to 4096 in the Osirix DICOM viewer within 20 time points during one cardiac cycle. The mean of the measured greyscale values within the region of interest in each time point was exported with the extension “ROI export” of the Osirix program. The measured mean grey value was divided by 4096 and subsequently multiplied by 2 cm/s (velocity encoding of the phase contrast sequence) for calculation of the velocity.

Parameters of interest within one cardiac cycle included amplitude of the velocity signal ranging from the maximum negative to the maximum positive peak, maximum cranial respectively maximum caudal velocity, and the area under the curve (AUC) of the movement signal. AUC was calculated by stepwise summation of calculated squared areas in each time point (1/20 RR time multiplied by the measured velocity). Negative velocity values were transformed to a positive value for calculation of the area under curve.

### Phase drift correction of spinal cord motion measurement

As phase contrast imaging is a relative measure of motion a so-called “phase drift”^10^ leads to an offset error of the raw data ending in misleading velocity values (Fig. 1c). Therefore, phase drift conducts to an over- or underestimation in the velocity measurements and a correction for phase drift is needed. In our analysis phase drift correction was done by subtraction of the mean of all 20 velocity measurements within one cardiac cycle from the raw velocity value at each time point (Fig. 1d), as net motion of the spinal cord over one cardiac cycle is assumed to be 0 (start and end location of the spinal cord is expected to be at the same position - assuming mean velocity has to be 0).

### Statistical analysis

Statistical analysis was conducted using SPSS (IBM, Version 23). Metrics are reported as group mean ± SD. Between segment differences were calculated using the Friedmann test. For reliability analysis intraclass correlation coefficients (ICC) were calculated (two way mixed model, absolute agreement, average measures). For bivariate correlations Spearman Rho coefficients were calculated. Significance level of alpha was set to <0.05.

### Inter-rater reliability

Inter-rater reliability was assessed by calculating intraclass correlation coefficients (two-way mixed model, absolute agreement, average measures). Two independent raters placed the ROI midcentred into the spinal cord as explained above (velocity calculation) in all cervical segments of each healthy participant (n = 18).

### Test-retest reliability

For evaluation of test-retest reliability 9 of the 18 healthy volunteers were scanned a second time after 732+/− 164 days. Velocity measurements were done by the same rater at the first and second scan and intraclass correlation coefficient (two-way mixed model; absolute agreement; average measures) were calculated.

## Results

### Subject Characteristics

Systolic and diastolic blood pressure (n = 17) were within normal limits before (129.7 ± 13.7/80.0 ± 7.3 mmHg) and after the MRI scan (122.7 ± 10.5/77.0 ± 7.7 mmHg). Mean value of blood pressure before and after measurement was systolic 126.2 ± 11.7 mmHg, diastolic 78.5 ± 7.2 mmHg with a mean arterial pressure of 94.4 ± 8.2 mmHg.

### Anatomical data

In sagittal T2 images spinal canal anterior-posterior (ap) size was 1.11–1.29 cm. In axial images ap diameter ranged from 1.29–1.58 cm, right-left (rl) diameter from 2.03–2.27 cm. Spinal canal cross sectional area (CSA) was 2.21–3.20 cm^2^, spinal cord CSA 0.56–0.83 cm^2^. The apex of the cervical spine was observed at disc 5/6 in 7, disc 6/7 in 4, vertebra C6 in 3, vertebra C5 in 2 and disc 4/5 respectively vertebra 4 in 1 patient.

### Spinal cord motion pattern

**Figure 2:**
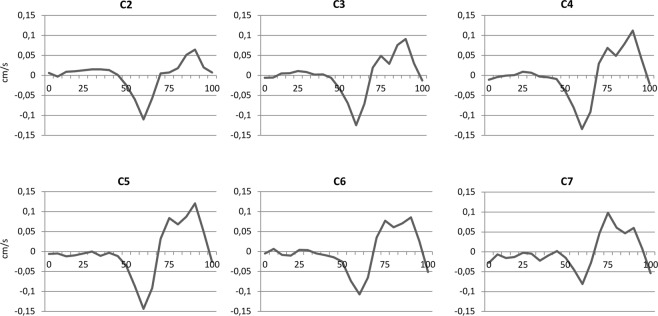
Spinal cord motion in each cervical segment during one heartcycle. Velocity signal (cm/s) was collected in 18 healthy volunteers. The curves represent the group mean of corrected values in 20 timepoints during one heartcycle (0–100%) triggered by peripheral pulse signal. Over all segments, a cranio-caudal followed by a caudo-cranial movement of the spinal cord could be observed. First half of the heartcycle showed nearly no motion in all segments.

A characteristic, comparable motion pattern over all cervical segments could be observed (Fig. 2), with nearly no motion in the first half of the heart cycle (measured by peripheral pulse triggering), followed by a cranio-caudal and afterwards caudo-cranial oscillatory movement.

### Phase drift correction

In our measurements a positive “phase drift” about 0.2–0.3 cm/s could be observed in all participants, which would have led to a misleading systematic overestimation of spinal cord motion (Supp. Fig. 1). By subtracting the mean velocity of all 20 time points from the raw value at each single time point in each segment, motion signal over one heart cycle could be sufficiently corrected (Fig. 2).

### Interrater reliability

The inter-rater reliability of the 18 baseline measurements between two independent raters was with ICCs of almost 1 (p < 0.001) excellent in all cervical segments (Table 1).

**Table 1:** Inter-rater reliability.

	Amplitude		Max cranial		Max caudal		Displacement	
	ICC	p	ICC	p	ICC	p	ICC	p
C2	0.999	0.000	0.998	0.000	0.999	0.000	0.998	0.000
C3	0.999	0.000	0.999	0.000	0.999	0.000	0.999	0.000
C4	1	0.000	0.999	0.000	1	0.000	0.999	0.000
C5	1	0.000	0.999	0.000	0.999	0.000	0.999	0.000
C6	0.998	0.000	0.997	0.000	0.998	0.000	0.996	0.000
C7	0.992	0.000	0.980	0.000	0.993	0.000	0.998	0.000

For inter-rater reliability between two independent raters intraclass correlation coefficients (two-way mixed model, absolute agreement, average measures) were calculated for corrected velocity values in each cervical segment for amplitude, maximum cranial and caudal velocity and for the displacement.

max = maximum, ICC = intraclass correlation coefficient.

### Test-retest reliability

ICCs demonstrated excellent test-retest reliability for all parameters at C2 to C6 (ICC = 0.766–0.964; p = 0.000–0.038), but not for max cranial velocity at C6 and all parameters at C7 (ICC = 0.220–0.604; p = 0.103–0.384) (Table 2). ICCs of raw (not corrected for phase drift) values showed inferior test-retest reliability, i.e. for displacement measurements (ICC = 0.171–0.685; p = 0.020–0.216), which were not reliable.

**Table 2:** Test-retest reliability.

	Amplitude		Max cranial		Max caudal		Displacement	
	ICC	p	ICC	p	ICC	p	ICC	p
C2	0.855	0.008	0.822	0.016	0.867	0.006	0.815	0.001
C3	0.868	0.007	0.838	0.012	0.877	0.006	0.858	0.002
C4	0.866	0.011	0.873	0.009	0.839	0.018	0.852	0.010
C5	0.946	0.001	0.908	0.002	0.879	0.008	0.968	0.000
C6	0.756	0.038	0.586	0.103	0.856	0.013	0.781	0.009
C7	0.678	0.117	0.604	0.124	0.220	0.384	0.604	0.134

For test-retest reliability intraclass correlation coefficients (two-way mixed model, absolute agreement, average measures) between two measurements were calculated for corrected velocity values in each cervical segment for amplitude, maximum cranial and caudal velocity and for the displacement.

max = maximum, ICC = intraclass correlation coefficient.

### Measured spinal cord motion values

The mean amplitude of spinal cord motion ranged from 0.296 ± 0.088 cm/s in segment C2 to 0.445 ± 0.163 cm/s at level C5 (Table 3). Maximum cranial velocity ranged from 0.115 ± 0.041 cm/s at C2 to 0.186 ± 0.078 cm/s at C5. Maximum caudal velocity ranged from −0.169 ± 0.055 cm/s in segment C7 to −0.259 ± 0.094 cm/s at C5. For the displacement, the highest values could also be observed at C5 (0.054 ± 0.016 cm), with lowest values at C2 (0.036 ± 0.009 cm). Raw, uncorrected values are provided in Supplementary Material for comparison (Supp. Table 1).

**Table 3:** Segmental spinal cord motion readouts.

	Amplitude		Max cranial		Max caudal		Displacement	
	Mean	SD	Mean	SD	Mean	SD	Mean	SD
C2	0.296	0.088	0.115	0.041	−0.182	0.060	0.036	0.009
C3	0.335	0.108	0.132	0.042	−0.204	0.072	0.042	0.011
C4	0.387	0.139	0.161	0.052	−0.226	0.096	0.048	0.013
C5	0.445	0.163	0.186	0.078	−0.259	0.094	0.054	0.016
C6	0.358	0.095	0.161	0.050	−0.197	0.061	0.049	0.010
C7	0.339	0.083	0.168	0.053	−0.169	0.055	0.051	0.015

Values of spinal cord motion were calculated for corrected velocity values in each cervical segment for amplitude (cm/s), maximum cranial (cm/s) and caudal velocity (cm/s) and for the displacement (cm).

max = maximum, SD = standard deviation.

### Intersegment differences

**Figure 3:**
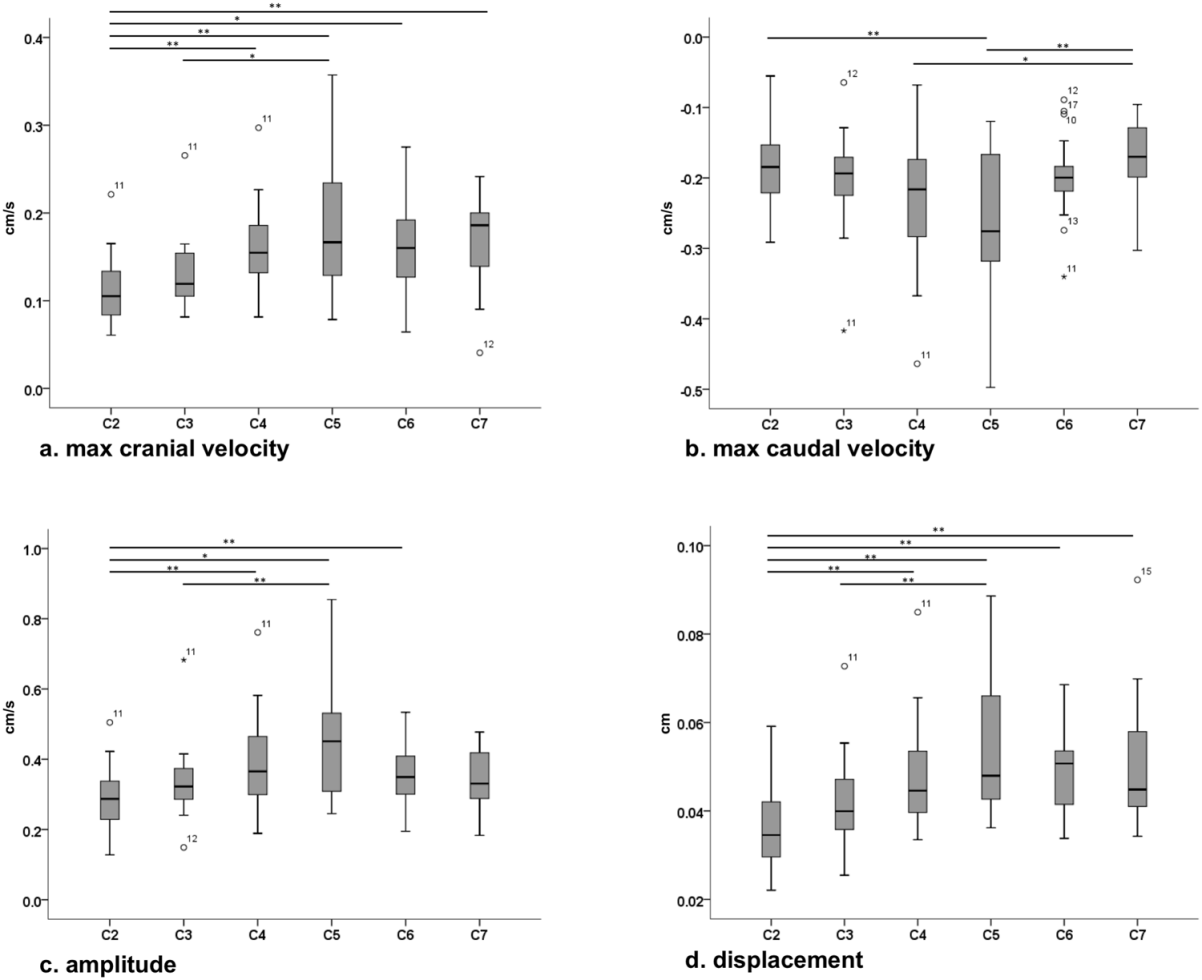
Intersegment differences of spinal cord motion. Corrected values of maximum cranial velocity (**a**), maximum caudal velocity (**b**), amplitude (**c**) and displacement (**d**) are shown for each segment. Significant differences are marked with bars and could be observed between several segments for all parameters. cm/s respectively cm; max = maximum; *p < 0.05; **p < 0.01.

Significant amplitude differences were found between C2–C4 (p = 0.015), C2–C5 (p < 0.001), C2–C6 (p = 0.049) and C3–C5 (p = 0.009) (Fig. 3c). For maximum cranial velocity significant differences were observed between C2–C4 (p = 0.005), C2–C5 (p < 0.001), C2–C6 (p = 0.020), C2–C7 (p = 0.004) and C3–C5 (p = 0.049) (Fig. 3a). For maximum caudal velocity, differences could be found between C2–C5 (p < 0.001), C4–C7 (p = 0.049) and C5–C7 (p < 0.001) (Fig. 3b). For the displacement significant differences were observed analogue to maximum cranial velocity (C2–C4 (p = 0.001), C2–C5 (p < 0.001), C2–C6 (p < 0.001), C2–C7 (p = 0.001), C3–C5 (p < 0.001); Fig. 3d).

### Correlations of spinal cord motion to anatomic measures

**Figure 4:**
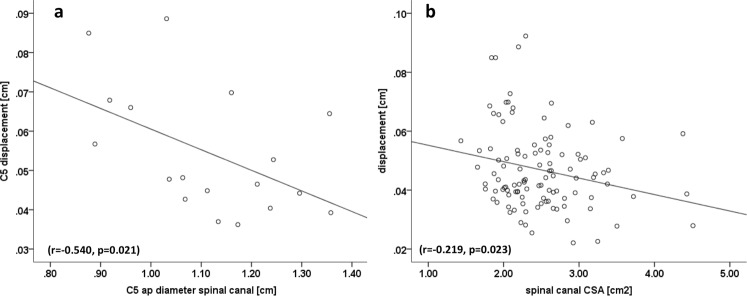
Correlations of spinal cord motion to anatomic measures. Displacement was correlated to anterior-posterior spinal canal diameter at C5 in sagittal imaging (a; r = −0.540, p = 0.021) and to spinal canal cross sectional area over all segments (b; r = −0.219, p = 0.023). CSA = cross sectional area.

Greater spinal cord displacement at cervical level C5 was correlated with smaller anterior-posterior diameter in sagittal imaging (r = −0.540; p = 0.021) (Fig. 4a). Considering spinal cord motion across all cervical segments, higher displacement correlated with a smaller spinal canal CSA (r = −0.219; p = 0.023, Fig. 4b), smaller spinal canal ap diameter (axial image: r = −0.191; p = 0.048, sagittal image: r = −0.208; p = 0.030, plot not shown) and smaller CSF space (r = −0.195; p = 0.044, plot not shown). According to spine configuration, the apex was mostly at disc level C5 (n = 7), followed by disc C6 (n = 4). No correlation between the apex of the spine and motion amplitude (r = −0.093; p = 0.713), displacement (r = 0.211; p = 0.401), max cranial (r = −0.002; p = 0.995) or caudal (r = −0.171; p = 0.497) velocity was found. The same was true for the angle of the spine configuration (amplitude: r = 0.204; p = 0.417; displacement: r = 0.012; p = 0.964; max cranial velocity: r = −0.217; p = 0.386; max caudal velocity: r = −0.171; p = 0.497).

### Correlations of spinal cord motion to subject characteristics

**Figure 5:**
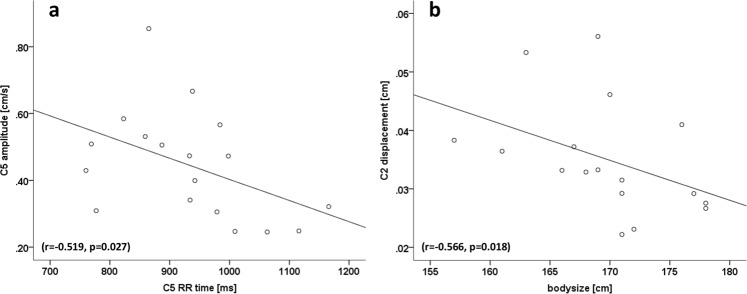
Correlations of spinal cord motion to subject characteristics. Smaller spinal cord motion amplitude at C5 correlated to longer RR time (a; r = −0.519; p = 0.027). Higher spinal cord displacement at C2 correlated to smaller bodysize (b; r = −0.566; p = 0.018).

Longer RR time of heart cycle, reflecting slower heartbeat, was correlated to smaller amplitude at C3 (r = −0.513; p = 0.030; plot not shown), C4 (r = −0.624; p = 0.006; plot not shown), C5 (r = −0.519; p = 0.027; Fig. 5a) and C6 (r = −0.501; p = 0.034; plot not shown). The same was true for max cranial velocity at C4 (r = −0.664; p = 0.003) and C6 (r = −0.492; p = 0.038; plots not shown). Longer RR time was correlated to less negative values of max caudal velocity, reflecting decreased downwards motion at C3 (r = 0.492; p = 0.038) and C4 (r = 0.598; p = 0.009; plots not shown).

Higher displacement at C2 correlated with smaller body size (r = −0.566; p = 0.018; Fig. 5b) and smaller mean arterial pressure (r = −0.487; p = 0.047; plot not shown). At C3 (r = −0.512, p = 0.036) and C5 (r = −0.523, p = 0.031) a correlation between higher displacement to lower systolic blood pressure after imaging was observed (plots not shown). Sex and body weight were not correlated with any readout.

## Discussion

This study is the first measuring physiological spinal cord motions across all cervical spinal segments. Physiological, pulse-synchronic oscillations of the spinal cord have been observed and described in prior studies^2,5,18–22^, but mainly focused on individual stenotic and adjacent cervical segments in DCM patients. In our healthy study cohort, comparable motion patterns in all cervical segments could be observed with nearly no motion in the first half of the heart cycle (measured by peripheral pulse triggering), followed by biphasic cranio-caudal oscillatory movement (Fig. 2). This finding corresponds to studies observing analogue cervical spinal cord motions (first caudal and then cranial movements) using ECG triggering^2,5^.

All spinal cord motion readouts except amplitudes are highly influenced by phase drift of MRI measurements. Phase drift was reported to differ between scanners and also between different time points in the same scanner^10^. Phase drift correction was conducted by phantom measurements in earlier MRI studies^2,5,18^, but is time consuming and less applicable in a clinical setting. Also measurements of static tissue next to the spinal canal were used for calculation of the background velocity reflecting phase drift^18^. However, measuring static tissue was not reliable in our study between two different raters (data not shown). Vavasour *et al*.^6^ adjusted velocity curves by adding or subtracting a uniform velocity from each cardiac time point to make the net motion over a cardiac cycle equal to zero but did not report how this value was obtained. In other studies assessing spinal cord motions no systematic correction for phase drift was mentioned^4,7^, while Chang *et al*.^4^ did not need to correct as they used the half amplitude of the velocity signal, not altered by phase drift.

We propose phase drift correction by subtracting the the mean of all velocity values within one heart cycle from each single value, assuming oscillatory spinal cord motion starts and ends at the same position and net motion is equal to 0. The same assumption was reported before^6^, but no systematic calculation for correction was provided. Using this simplified calculation, sufficient correction could be shown in all measurements. Most important, the amplitude of the velocity signal is not affected by the phase drift and may therefore be an attractive parameter for future studies on spinal cord motion as no correction is needed.

Most studies did not report on reliability of spinal cord motion measurements^2,4–6,18^. In our study interrater reliability between two independent raters was excellent using a predefined ROI placed mid-centred into the spinal cord for all parameters at all levels. These results are in line with prior reported good inter-rater reliability using a smaller region of interest (19.68 mm^2^), also mid-centred into the spinal cord^7^. Using a bigger ROI in our study further improved inter-rater reliability.

Our study is the first to report the test-retest reliability in phase contrast spinal cord motion measurements. Even though second measurements were performed in mean after two years (732+/− 164 days) in stable healthy controls test-retest reliability remained excellent in all segments except at C7, using the proposed phase drift correction. However, applying raw values without correction test-retest reliability was inferior, i.e. the displacement values were not reliable. Phase drift can differ between two measurements^10^, ending in different absolute raw values and low test-retest reliability, underlining the importance of systemic phase drift correction. Poor reliability at C7 might be mostly attributed to inferior signal to noise ratio in this segment as distance to the head coil in the MRI scanner is high^23^. Due to sufficient phase drift correction in the two different measurements, the proposed method should also be sufficient in different scanners with different phase drift^10^, but this was not evaluated in the present study.

Parameters of interest used in prior analyses were amplitude^4^, total displacement^6,7^, maximum displacement^6^, peak velocities^2,22^, mean velocity^6^ and velocity difference between cervical segments^5^. However, due to different readouts comparability between measurements is limited. We introduce a standardized MRI evaluation, including a phase drift correction, ending up in four different parameters (amplitude, max cranial respectively max caudal velocity, displacement). Wolf *et al*.^7^ did not correct for phase drift and therefore a overestimation of velocity measures cannot be excluded. Chang *et al*.^4^ reported a mean value of cord motion of 5 ROIs at vertebral body level C2 to C6 of 0.28 cm/s for the half peak to peak amplitude, while no individual segmental values were reported. As significant differences could be shown between these segments in our study, calculating a mean appears not reasonable. Vavasour *et al*.^6^ reported at C5 disc level a mean cord velocity of 0.144 cm/s and a displacement of 0.133 cm in controls, comparable to a mean velocity of 0.188 cm/s respectively a displacement of 0.176 cm in our uncorrected raw data. A correction for phase drift is mentioned in their study, but no detailed information was provided.

Impact of different confounders on spinal cord motion might explain discrepancies between reported velocity values along different studies. Magnetic field strength might influence accuracy of measurements. As initial MRI measurements were performed using lower field (0.5–1.5T) strength^2,5,18,22^, recent studies mostly measured at higher field (3T) MRIs^4,6,7^. Additionally, the influence of the chosen velocity encoding for the phase contrast MRI sequence on accuracy of motion measurements remains open. In our and some prior studies^5,18^ an encoding of 2 cm/s, focusing on slow spinal cord motion was implemented, in contrast to 5 cm/s^2,7^ and 6 cm/s^4^ in other studies. As MRI digital imaging and communications in medicine (DICOM) data system encodes for 4096 grey values only, higher velocity encoding might end in lower accuracy of measurements of slow velocities like in spinal cord motions. Therefore, velocity encoding should be adapted as far as possible to expected velocity values.

Also the shape and the size of the region of interest will influence velocity values as they are collected as a mean value within the ROI. On the one hand it should cover a large part of the spinal cord, but partial volume effects at the spinal cord - CSF border should be avoided, and the size should be feasible to be used in stenotic segments with the cord being squeezed (i.e deformed). In contrast to our ellipsoid ROI of 30,52 mm^2^, prior studies used a rectangle ROI of 19.68 mm^2^ ^7^, a manually drawn ROI around the spinal cord^6^, a round ROI covering the ap diameter of the spinal cord^4^, a circular ROI^2,5^ with no information about the size and also differing sizes between patients in manually drawn ones^4,6^.

Influence of age on spinal cord motion, as measurements were performed in younger volunteers before^2,4,5,7,18^ remains unknown. In our population no relation of spinal cord motion to age could be found, but all participants were older than 50 years comparable to only one prior study^6^.

In physiological conditions, the origin of spinal cord motions was mostly attributed to remote factors like intracranial CNS- and CSF-pulsation within the cardiac cycle^5^ and breathing^24^. Breathing was not assessed in our study, but relation of higher spinal cord motion to lower RR time respectively higher heart rate could be observed. Interestingly studies assessing pulse wave velocities and blood flow in blood vessels showed also about 20% higher values with increasing heart rate^25–28^, underlining the impact of arterial pulsation on spinal cord motion. Additionally, focal influences on spinal cord motion due to expansion of segmental arteries could be shown in a canine model as oscillations diminished after transection of the local vascular support^21^. However, association of higher spinal cord motion to lower blood pressure in our measurements is contradicting previous findings in arterial blood vessels^29,30^ and remains unexplained.

Intersegment comparisons in our study revealed significant differences between various cervical segments. Highest spinal cord motion could be observed at C5. No systematic evaluation of intersegment differences is provided in most prior studies^2,4,6,22^, or only in patients between compressed and non-compressed segments^4,5^. Wolf *et al*.^7^ report a displacement ratio between C2 and C5 of 1 in healthy controls, reflecting no differences between motions in these segments, but uncorrected, potentially overestimated values were used for calculation. Max spinal cord motion was also reported at C2 and C5 in the latter study, but no statistical analysis about significant differences between these segments, as only differences to patients were evaluated.

Spinal cord motion was negatively correlated to spinal canal measures. These results are in line to the law of Hagen–Poiseuille^31^. The relation to anatomic conditions is comparable to reports in CSF dynamics before^32,33^. Specifically CSF velocity was higher in smaller spinal canal and smaller subarachnoidal space also with a maximum at C5^32,33^, but no relationship of spinal cord motion to anatomic conditions could be found in prior evaluations in healthy controls^6,7^. Additionally, a correlation between spinal cord and CSF motion at C2 was reported before^7^. As CSF-flow was frequently discussed to influence spinal cord motion^1,34–37^, our results underline the close interaction between CSF and spinal cord dynamics, with highest motion values at C5 either. Due to low interrater reliability of CSF measurements on level of stenosis in DCM patients^7^ and low velocity encoding used in our study no CSF assessments were done.

Correlation of higher spinal cord motion to smaller body size in our population has to be attributed to smaller anatomic conditions in smaller people, as correlations of smaller body size to smaller anatomic measures, i.e. spinal canal could be observed (data not shown).

The configuration of the spine, i.e. the apex and the angle did not influence spinal cord dynamics in our population. Also no relation to weight and age could be observed. No prior evaluation on this could be found in literature.

Interestingly the C5 level exhibited the highest spinal cord motion and this level is also one of the most affected by spinal canal stenosis and in DCM^34^. Increased spinal cord motion, especially as shown in DCM patients^4–7^ might reflect increased mechanical stress to the spinal cord conducting to damage and deterioration.

Different MRI techniques have been applied to evaluate the spinal cord complementary to standard clinical sequences, aiming for additional information about the cords tissue integrity. Most recently a multimodal MRI protocol in DCM was introduced^38,39^. The protocol including diffusion tensor imaging (DTI), magnetisation transfer ratio (MTR), T2* white matter/grey matter contrast ratio and T2 cross sectional area measurements at, below and above the level of the spinal stenosis shows promising ability to reveal spinal cord damage and deterioration over time, even in asymptomatic patients^40,41^. However, these advanced microstructural measurements require extensive postprocessing, and still await to be implemented in the daily clinical workup, while spinal cord motion measurements can be easily evaluated. Also, these MRI sequences are more time consuming compared to phase contrast measurements. In addition, increased spinal cord motion might indicate the risk of spinal cord damage due to increased mechanical stress, even if evident microstructural damage could not yet be observed. Therefore, it might be able to identify high risk patients and could help to decide for decompressive surgery.

## Conclusion

This study provides promising data for a standardized evaluation and corrections for systemic bias in phase contrast imaging and further clinical implementation of spinal cord motion. The proposed protocol is applicable to develop normative values (common or per site) of cranio-caudal cervical spinal cord motions, to discern and monitor pathologic conditions and its association to DCM where spinal cord motion is influenced by spinal canal size, heart rate, body size and blood pressure.

## Supplementary information

Supplementary information

## Data Availability

The datasets generated and analyzed during the current study are available from the corresponding author on reasonable request.
