# Supplementary material for: Segmental differences of cervical spinal cord motion: advancing from confounders to a diagnostic tool
Source: Sci Rep. 2019 May 15;9:7415. doi: 10.1038/s41598-019-43908-x (PMC6520379; doi:10.1038/s41598-019-43908-x)
Supplement: Supplementary file 1 — Supplementary information [file 41598_2019_43908_MOESM1_ESM.pdf]

Supplementary information

Segmental differences of cervical spinal cord motion: advancing from confounders to a diagnostic tool

Hupp M[1*], Vallotton K[1], Brockmann C[1], Huwyler S[1], Rosner J[1], Sutter R[2], Klarhoefer M[3], Freund P[1], Farshad M[4], Curt A[1,4]

1 Spinal Cord Injury Center, Balgrist University Hospital, Zurich, Switzerland

2 Radiology, Balgrist University Hospital, Zurich, Switzerland

3 Siemens Healthcare AG, Zurich, Switzerland

4 University Spine Centre Zurich, Balgrist University Hospital, Zurich, Switzerland

*corresponding author

Hupp Markus

University Hospital Balgrist

Spinal Cord Injury Centre

Forchstr. 340

8008 Zurich

Switzerland

Email: Markus.Hupp@gmx.de

Phone 0041-44-386-3905

	amplitude		max. cranial		max. caudal		displacement	
	mean	SD	mean	SD	mean	SD	mean	SD
**C2**	0.296	0.088	0.328	0.050	0.032	0.063	0.186	0.033
**C3**	0.335	0.108	0.340	0.045	0.005	0.081	0.185	0.032
**C4**	0.387	0.139	0.358	0.046	-0.031	0.106	0.181	0.030
**C5**	0.445	0.163	0.374	0.082	-0.071	0.099	0.176	0.035
**C6**	0.358	0.095	0.339	0.053	-0.019	0.068	0.165	0.040
**C7**	0.339	0.083	0.365	0.062	0.026	0.100	0.181	0.063

Supp. table 1 Uncorrected segmental spinal cord motion readouts

Values of spinal cord motion were calculated with the collected raw velocity values in each cervical segment without correction for confounders. Displayed are the values for the amplitude (cm/s), maximum caudal velocity (cm/s), maximum cranial velocity (cm/s) and for the displacement (cm).

max = maximum, SD = standard deviation

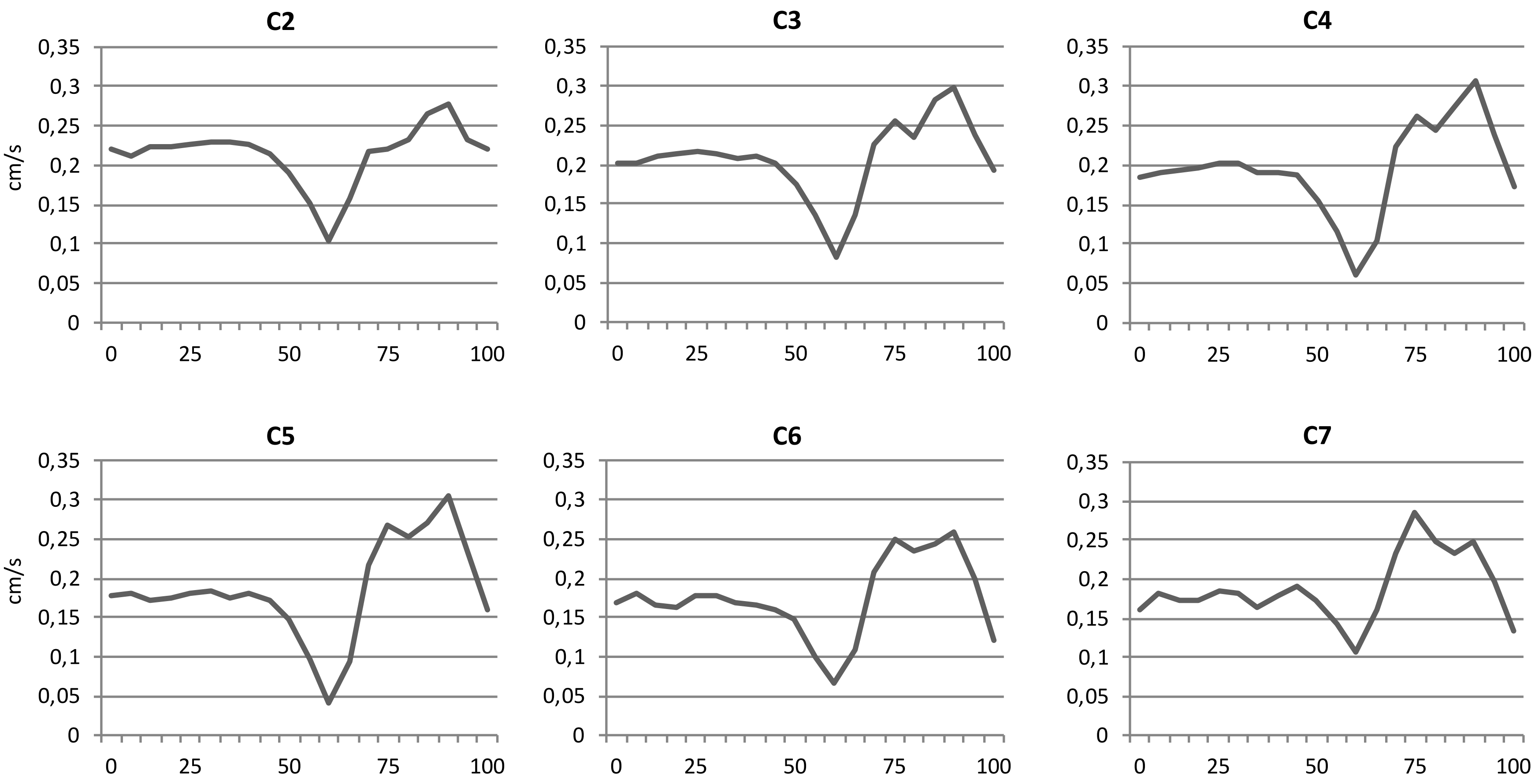

Supp. figure 1 Uncorrected spinal cord motion signal in each cervical segment during one heartcycle
Velocity signal (cm/s) was collected in 18 healthy volunteers. The curves represent the group mean of uncorrected raw values in 20 timepoints during one heartcycle (0-100 %) triggered by peripheral pulse signal. A positive “phase drift” respectively offset error with misleading high velocity values could be observed in all segments.
